# Parents’ experiences of caring for a child with an eating disorder: the impact of financial challenges

**DOI:** 10.1186/s40337-025-01278-y

**Published:** 2025-09-26

**Authors:** Hannah Shaw, Nadia Ranceva, Dawn Langdon

**Affiliations:** 1 Royal Holloway University of London, Egham Hill, Egham, UK; 2 The Eating Disorder Young Person’s Service, Alder Hey Children’s Hospital Foundation Trust, Liverpool, UK

**Keywords:** Eating disorders, Financial impact, Caregiver burden, Young people, Parents, Family caregiving, Socioeconomic Factors

## Abstract

**Background:**

Research has highlighted significant challenges associated with caring for a child or adolescent with an eating disorder, and that the impacts on the family are both long-standing and widespread. Within the literature, parents have reported that all aspects of their lives were affected, including their occupational functioning and financial situation. This study explored parents’ experiences of the challenges associated with caring for a child with an eating disorder, focusing on the financial implications and the effect of these on the family and treatment.

**Methods:**

Using the exploratory descriptive qualitative approach, semi-structured interviews were completed with 12 parents recruited from a child and adolescent eating disorder service. Interview transcripts were analysed using reflexive thematic analysis.

**Results:**

The overarching theme generated was *Life is completely different living with the eating disorder*. The analysis process yielded nine sub-themes, which were grouped into three main themes: (1) *Financial costs*, (2) *Financial impact on us*, and (3) *Things that helped.* Parents reported significant interrelated psychosocial and economic impacts, which were exacerbated by their child’s resistance to treatment, an unpredictable course of illness, and a lack of understanding about eating disorders at a societal level.

**Conclusions:**

The findings suggest financial pressures exacerbate the significant caregiver burden and, without adequate financial support, some families struggle to afford essential treatment costs, both of which can impede recovery. Thus, services must address the financial implications and each family’s unique situation throughout the assessment and treatment process. Services can mitigate burdens for parents by tailoring support to families’ needs, offering foodbank vouchers, providing information on benefits, and assisting with reclaiming parking and public transport costs. Further research is required to assess the extent of the financial burden and its impact on treatment, and to determine which individuals are most affected.

## Abstract (plain language summary)

Research has found that there are significant challenges faced by families caring for individuals with eating disorders, including financial challenges. This study interviewed parents who were caring for a child with an eating disorder to better understand the financial implications and the effect of these on the family and treatment process. The findings highlight that financial costs contribute to caregiver burden, and without support, some families may not be able to afford all components of treatment, which can negatively impact recovery. Practical and easy-to-implement strategies are provided, for both parents and services, to reduce the negative impact of financial challenges on families and ED recovery.

## Background

Around 8% of the global population will be affected by an eating disorder (ED) in their lifetime [1]. Since the COVID-19 pandemic, there has been a significant increase in the incidence and severity of ED pathology worldwide [2, 3], with the impacts being most severe for young people [4, 5]. Between 1998 and 2020, National Health Service (NHS) hospital episodes in England increased by 168% for adults and 361% for children [6]. Figures suggest treating the psychological symptoms of an ED costs the NHS around £7,200 per patient annually, covering inpatient care, outpatient care, primary care and family therapy [7]. These figures do not include additional service involvement for associated physical health complications [8]. Inpatient treatment is predicted to account for around 75% of healthcare costs for young people with Anorexia Nervosa [9], highlighting the importance of promoting and facilitating recovery at home. In addition to the economic argument, evidence suggests ED outcomes are better when patients are cared for at home [10].

The role of the family in recovery has substantial empirical support, and family-based treatment (FBT) is the leading first line approach for young people in guidelines worldwide [11, 12]. For over half of young people diagnosed, their ED will persist for over five years, and approximately one-third will never fully recover [13]. Thus, there are significant protracted impacts for families and carers [14, 15]. Family caregivers of people with EDs report a significantly poorer quality of life, as well as feelings of sadness, powerlessness, and desperation [16]. In addition to affecting family functioning and carers’ psychological well-being [17, 18], ED caregiving has been associated with substantial financial challenges [5, 19]. Despite receiving ED treatment that is free at point of use in the UK, estimates suggest people with EDs and their carers face annual costs of up to £11,000 and £9000 respectively [7]. These totals are a combination of direct financial costs incurred as a result of the ED (e.g., for treatment and travel to treatment) and loss of income due to taking time off work or education.

An Australian study found 97% of adults receiving ED treatment faced economic hardship and 18% reported cost-related nonadherence, meaning they had either missed a medical appointment or failed to fill a prescription due to cost [20]. Higher out-of-pocket expenditure and cost-related nonadherence were associated with poorer health and quality-of-life, and a more threatening perception of the illness [20]. Financial factors have also been cited as a barrier to accessing ED treatment [5, 21, 22] and were the main reason for not seeking help amongst ethnic minority groups in the US [23]. Patients who wait longer to access ED treatment are more likely to drop out of interventions [24] and show less improvement in symptomology [25]. Early intervention is associated with better outcomes and improved course of illness [26]. Thus, reducing barriers to seeking, accessing, and adhering to treatment is critical; especially as previous estimates have suggested only 23% of people with EDs in England access support [27].

The Darzi Report [28], an independent investigation of the NHS in England, emphasises equitable access to support and the reduction of disparities in healthcare outcomes as key priorities for the ten-year health plan. This report also highlights the importance for healthcare services to increase their awareness of factors that perpetuate health inequalities [29]. Despite previous research findings that financial factors associated with EDs were a source of distress for families [30] and contribute to caregiver burden [31], there has been limited understanding of the specific costs involved. Although recent studies have quantified the financial impacts [5, 19], there is still little insight into how these costs are managed and experienced by families and services—particularly in countries where healthcare is free at the point of use. The present study aims to address a gap in the literature by qualitatively exploring the experiences of parents to better understand the impact and management of costs associated with caring for a child with an ED. It seeks to develop awareness and identify where support may be required to reduce barriers to ED treatment, and mitigate negative impacts on families, by informing clinical practice, policy, and service development.

## Methods

### Design

The study adopted an exploratory descriptive qualitative approach (EDQ), as it has been identified as an appropriate theoretical framework for exploring and describing participants experiences and studying areas of healthcare practice that are under researched [32].

#### Expert-by-Experience (EBE) involvement

A parent group within the ED service was first consulted to discuss the research idea and assess its relevance, benefit, and feasibility. Once confirmed, two EBEs were consulted individually to develop the information sheet, consent form, interview schedule, and plans for dissemination.

#### Ethics

Prior to recruitment, Health Research Authority, Research Ethics Committee, and local research and development approvals were obtained. All participants received information on confidentiality and the right to withdraw before providing written consent online.

### Participants and sampling

A total of 12 participants were recruited from a young person’s ED service in the Northwest of England. Participants were parents of patients in the service at the time of recruitment who volunteered to take part. Information packs were distributed by reception staff and clinicians to eligible parents and an advertisement was displayed in the clinic. Parents interested in participating were asked to inform their ED clinician or the administration team.

Six to 10 participants are considered appropriate for small projects conducting interviews with a purposive sample [33]. An upper and lower sample size range of eight to 14 was set to allow the sample size to be shaped by reviewing the adequacy of the data to answer the research question [34].

#### Inclusion and exclusion criteria

To be included in the study, parents had to have a good level of spoken English and be a main carer for the young person with the ED, live with them, and be actively involved in their treatment. The young person must have been receiving treatment at the service for at least two months. As the study aimed to explore the financial implications and their impacts specifically, parents also had to self-identify as being financially impacted by their child’s ED, which allows for a purposeful sample that aligns with the EDQ approach [32]. Participants were excluded if involvement was deemed inappropriate by clinicians, based on their clinical judgement, for reasons such as safeguarding issues or high levels of distress.

### Data collection

#### Participant characteristics

All participants identifying as female (*n* = 9) referred to themselves as the patient’s mother, and all male identifying participants (*n* = 3) as the father. Children of participants had been in the ED service for an average of 18.6 months (*SD* = 8). Only two participants (P2 and P3) were parents of the same patient and interviewed together. See Table 1 for a detailed summary of participants characteristics.

Subjective descriptions of financial situation were collected, as research has shown that these descriptions more effectively predict health outcomes compared to objective indicators [35]. However, evaluations will likely be influenced by participants’ previous experiences of wealth [36]. Therefore, to ascertain how representative the current sample was of the general population of the area in which the service operates, participants were also asked about their accommodation status. Housing tenure is straightforward to collect and can be readily compared to census data. It is commonly used as an indicator of socioeconomic position within health research and is considered a key component of most people’s wealth [37]. Thus, both subjective and objective indicators were included to provide further contextual information to the reader [38], relevant to the generalisability of the findings. Accommodation status suggests that the sample was not representative of the wider population as 83.3% of participants owned their property, whereas the latest UK census indicates only 46.8% are homeowners in this area [39]. Thus, suggesting that on average the sample was wealthier than the area’s general population.

#### Interviews

Semi-structured interviews were conducted to facilitate exploration, via follow-up questions, of additional areas that emerged during interviews [40]. The interview schedule was informed by two pre-existing questionnaires from other studies investigating the financial impacts of EDs [7, 20], and discussions at a parent support group. This approach was taken to minimise the risk of overlooking aspects of the financial burden and to ensure the data were grounded in, and comparable with, existing literature. In response to the parent’s feedback, a general question regarding the overall impact of the ED was added at the beginning as it felt important to acknowledge and provide space to discuss the challenges of their ongoing experience. The draft schedule was then reviewed by a panel of experts and adjusted in line with feedback. Online interviews were conducted between October and December 2022, lasting an average of 36 min. Interviews were recorded and transcribed verbatim using transcripts generated by Microsoft Teams, then amended as necessary and anonymised by one of the authors.

**Table 1:** Participant information

	Gender	Months in ED service	Household composition	Accommodation status	Parents’ report of household financial situation
P1	Female	12	Mother, daughter (ED), younger daughter	Renting, private landlord	Quite difficult Very basic money
P2	Female	18	Mother, father, daughter (ED)	Own property	Comfortable
P3	Male	18	Mother, father, daughter (ED)	Own property	Comfortable
P4	Female	36	Mother, father, daughter (ED), 2 younger daughters	Own property	Stable, when working fulltime
P5	Female	10	Mother, father, daughter (ED), 3 younger daughters	Own property	Comfortable Not rich but do not struggle
P6	Male	15	Father, mother, daughter (ED), older daughter, younger daughter	Own property	Stable
P7	Female	20	Mother, father, daughter (ED), younger son	Own property	Get by / do not struggle
P8	Female	11	Mother, father, daughter (ED), younger son	Own property	Fairly comfortable
P9	Female	12	Mother, father, daughter (ED), older daughter, younger son, younger daughter	Own property	Comfortable
P10	Male	25	Father, mother, daughter (ED), older daughter	Own property	Comfortable
P11	Female	24	Mother, father, daughter (ED), younger son	Own property	Stable
P12	Female	15	Mother, father, daughter (ED), younger son, younger daughter	Tied Accommodation^1^	Very comfortable Employment not required

^1^ Tied accommodation refers to housing provided by an employer as part of the employee’s contract

### Analysis

Interview transcripts were analysed using reflexive thematic analysis (RTA), because it lends itself to the EDQ approach and is particularly well-suited to exploring individuals’ views and experiences while acknowledging the subjectivity of the researcher in the interpretation process [41]. The flexible framework of RTA allowed for an inductive and semantic approach to coding within a critical-realist framework, synonymous with the project aims [41]. A bottom-up, inductive approach was employed, in a recursive and iterative manner, to generate themes grounded in the data [41]. The six phases outlined and revised by Braun and Clarke [42–44] were used to guide the process.

Data familiarisation was undertaken, whereby transcripts were reread to facilitate immersion in the data, with notes entered in a reflective journal and cross-referenced with notes made during interviews. Codes were generated using NVivo (release 1.7.1) and evolved through repeated iteration, which was tracked to aid transparency and the process. The coded data was examined together and those sharing a similar concept were collapsed into a single item, before collating these into themes and sub-themes based on the researcher’s interpretation of shared meanings. Themes were reviewed recursively in relation to their compatibility with coded extracts, the entire data set, and the research question. Braun and Clarke’s [43] key questions and Patton’s [45] ‘dual criteria for judging categories’ were used to aid the process. A thematic map was created and revised throughout, and significant changes were recorded in the journal. Underlying data items were re-analysed to refine theme names and definitions and identify appropriate extracts for an illustrative write-up [46]. Themes and sub-themes were named using participants’ quotes that were deemed to effectively capture the content. A logical order was established so that themes were reported in a separate, yet meaningful, narrative that accurately represented the data.

#### Reflexivity

Interviews were conducted and analysed by the first author, a white British 29-year-old female trainee clinical psychologist of a working-class background, with experience in qualitative research and working clinically with young people with EDs and their parents. The first author has no children and no personal history of an ED, or experience of caring for someone with an ED. A reflective journal was kept and used alongside regular meetings with other authors to ensure reflective and reflexive practice was employed throughout [46]. The research team also comprised of a consultant psychiatrist, with direct clinical experience with this population, and an academic clinical psychologist, with no ED expertise.

#### Quality assurance

Elliott et al.’s [38] seven guidelines for qualitative studies were adhered to. Guidelines were met as follows: (1) use of a reflective journal and disclosure of the researcher’s background, theoretical stance, and assumptions; (2) provision of specific participant information; (3) inclusion of quotations for sub-themes; (4) consultation with the research team and participants, and through member reflections to enhance credibility [47]; (5) inclusion of a thematic map and narrative summary to illustrate themes and relationships between them; (6) recruitment extended in an attempt to increase sample variability and implications of underrepresentation are discussed; (7) findings presented in an accessible narrative which emphasised clinical applicability, and consultation of the target audience.

## Results

**Fig. 1:**
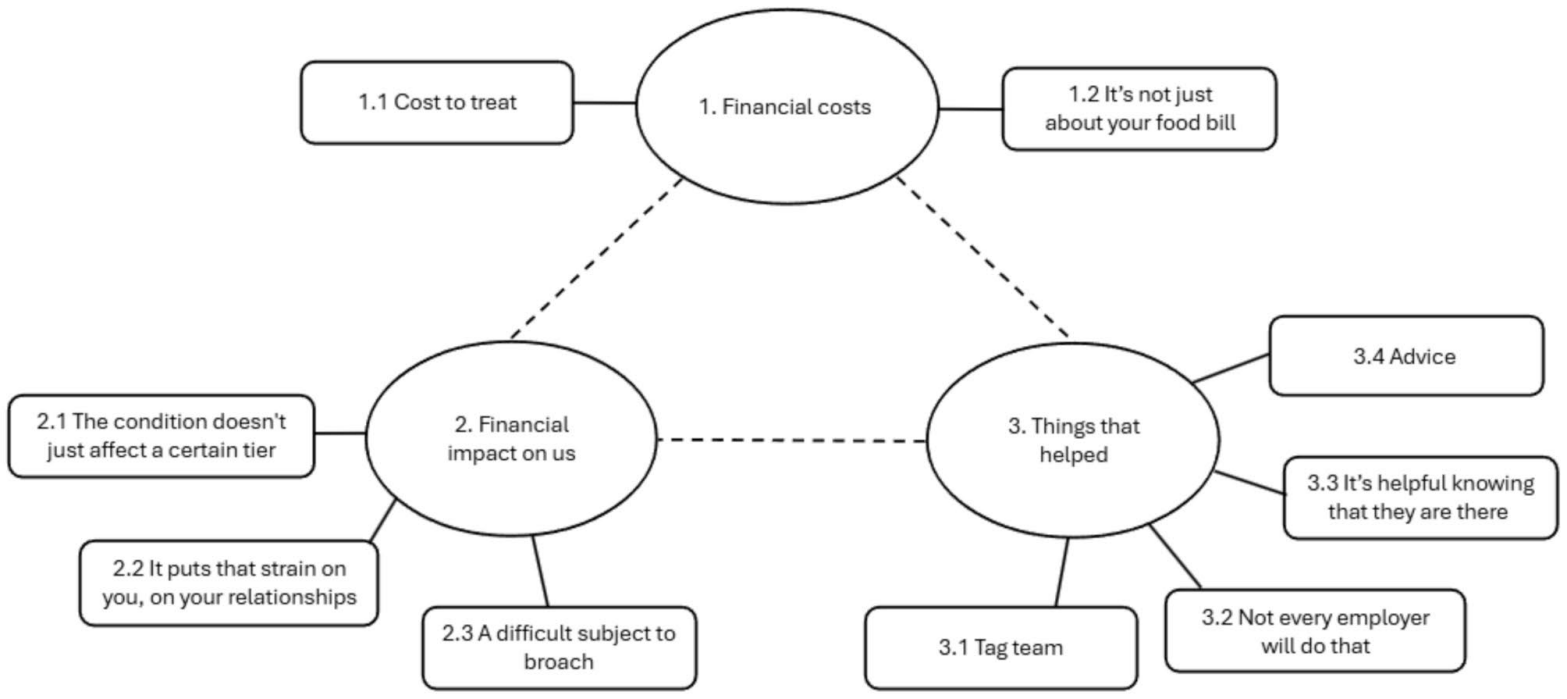
Thematic Map Illustrating Themes and Subthemes from RTA

The overarching theme generated from the data was *Life is completely different living with the ED*, which illustrates that caring for a child with an ED significantly impacted all aspects of participants’ lives. Parents discussed how resistance to treatment, an unpredictable course of illness, and lack of understanding at a societal level exacerbated the challenges. Many participants mentioned feeling isolated and a lack of support, including from their friends and General Practitioners (GPs). They also reflected on multiple aspects of their lives which were impacted, including relationships, family activities, well-being, financial situation, and their ability to work and socialise [48]. The RTA process yielded three main themes consisting of nine sub-themes (Fig. 1).

### Financial costs

This theme encapsulates the costs participants incurred as a direct result of their child’s ED. Many participants also reflected on fronting these additional expenses in the context of the escalating cost of living and widespread economic strain on households.

#### Cost to treat

Participants reported substantial increases in shopping bills due to needing larger quantities of food, and products they “wouldn’t have ordinarily bought” (P7), to meet the requirements of the standardised meal plan prescribed by the ED service. They were required to add products such as cheese, butter, and protein powder to food wherever possible, switch to full-fat options, and provide nourishing meals full of fresh produce. Participants expressed frustration around wasting considerable amounts of food, but stated they would willingly accommodate requests if it facilitated their child eating: “you overcompensate … and you will do that constantly because if she’s eating something, that [sic] that’s great” (P3). Participants also discussed spending a considerable amount of money on travelling to appointments: “the transport, sometimes three or four times a week to the hospital, was very, very expensive” (P1). Despite processes being in place for some individuals to reclaim public transport costs, these were not always well-advertised or accessible.

#### It’s not just about your food bill

Lost income was experienced by all participants, or partners: “it’s just a case of we just lose out at the moment till she gets better” (P9). However, the extent and impact of the loss differed, dependent upon job role, employer flexibility, and presence of a partner to share caregiving duties. Some participants were entitled to paid leave, or able to take unpaid leave, whilst others were unable to retain their job due to demands of the ED: “I couldn’t work. She needed me full time at home” (P1). Those that were self-employed were unable to take on as much work and others worked out-of-hours to avoid reductions in pay: “I was managing to maintain the output by doing [sic.], recovering the hours … they did suggest … to go down to a four-day week. But that wasn’t an option because of the impact it would have financially” (P6). Concerns were also raised regarding the impact of extended periods of leave on career progression, life satisfaction, and well-being. However, many participants noted that the work-from-home conditions permitted during the COVID-19 pandemic reduced the impacts and allowed them to retain income.

Participants reported more expensive energy bills due to increased use of heating to keep their child warm, crucial for those severely underweight: “If you’re being told that that’s [sic.] actually life threatening if she’s cold, you’d pull back somewhere else” (P11). This was intensified by long periods of not leaving the house to comply with treatment requirements of bedrest, reduced activity, or time off from school.

### Financial impact on Us

This theme conveys participants’ reflections on the consequences of the financial pressures stemming from their child’s ED.

#### The condition doesn’t just affect a certain tier

Participants’ experiences of affording ED-related expenses ranged from not having enough money to meet treatment requirements, to comfortably absorbing costs: “a far higher proportion of our income has been taken up” (P8). Ten participants considered themselves “fortunate” (P5) and expressed concern for those “living from pay-check to pay-check” (P3): “if it was just average salary then it would be a major problem” (P6). They also noted that the protracted duration of illness heightened the impact, and one participant (P10) estimated ED costs of around £3500 across two years, excluding additional food: “I don’t think generally, people would be aware that [sic.] that’s how long you’re paying hospitals … food, car parking, everything. It adds up massively” (P11).

Retaining income was considered imperative to coping with the financial burden, which posed difficult decisions for participants unable to work from home:

But with the household bills and everything being so high, and food is like a main contribution to that, it’s forced me back to work full time, so it’s affected [daughter’s] treatment in terms of I can’t be there. When I’m there with the meals, she’ll eat more. (P4)

There was consensus that participants would do whatever they could to fulfil treatment requirements and facilitate recovery: “We’d happily sell the house if she’d eat” (P8).

It’s absolutely heart-breaking. So if you feel that you are not fulfilling what they’re recommending to help recovery. That’s also then an additional thing that you have to battle …. But if we don’t, is she going to get better? (P11)

#### It puts that strain on you, on your relationships

Participants acknowledged the stress of encountering financial difficulties or having a seriously ill child, and then described their experience of confronting these challenges simultaneously: “you’re talking about such a like really stressful illness anyway …. you’re already heightened, and you’re already anxious … then you add in the financial pressure on top, it affects everybody’s mental health and family dynamics” (P4). Many said it caused “constant worry” (P5), voicing concerns about the rising cost-of-living, accumulating debt, losing their job, and taking time off: “…we probably were stressed. We probably were depressed” (P10). Seven participants reported a strain on their relationships caused by financial pressures, with some expressing that it resulted in conflict, debates, and disagreement within their household: “She was getting reduced pay … really stressing out about it, and we ended up arguing about it” (P10). One participant reflected on the potential impact of such tension on their child’s recovery process: “you’re supposed to work as a [sic.] as a unit together … but the friction between yourselves” (P4).

#### A difficult subject to broach

Participants had differing views on informing a clinician if they were struggling financially. Two participants who had already engaged in these conversations described the team as “helpful” (P9), and three others said they would feel comfortable to raise concerns if necessary. However, seven participants reported they would not inform a clinician for reasons including worries about discussing this in front of their child, believing the service could not assist, and concerns regarding their “privacy and pride” (P3). Links were also made to the shame and stigma they believed to be associated with not being able to provide for their child.

You don’t want to feel as a family that you [sic.] you’re kind of failing. (P4)

I’d feel a bit embarrassed if I couldn’t afford, you know, what’s basically a healthy diet. (P8)

I think there might … be an element of, urm, stigma attached. Not being able to afford to feed your kid properly. (P8)

Although the team initiated some conversations about financial help, participants noted it would be helpful to integrate this into the assessment and treatment process: “I think there needs to be, uh, some kind of structure, or way for families to feel comfortable, actually discussing that, without having to stand up first and say look I’ve got a problem. Because often you don’t” (P6). Where support had been discussed, clinicians offered food bank vouchers, forms to reclaim bus and taxi fares, car park tokens, and advice on benefits. For one participant, this advice was critical in being able to provide what was necessary for recovery: “at the beginning … I couldn’t afford it no, but they told me to apply for DLA [disability living allowance], for my daughter, so since we’ve had that it’s helped a lot, you know, to pay for food” (P1). Participants also relied on family for financial support, and other parents in the same situation, or forums, for advice.

There was consensus that there was a lack of financial support for families caring for a child with an ED:

No, there isn’t any, there’s nothing … It is so unfair because … your treatment is food. We can’t get [a] prescription for that, or you can’t get support for it. Urm whereas if you had a child with an illness, you get the medication, you get it free. (P4)

Participants felt there should be additional avenues of support for families struggling, such as supermarket vouchers, discounted energy bills, and the availability of a grant. Additionally, it was proposed that, at the beginning of treatment, services should provide information on what parents should expect, such as needing to take time off work and potential duration of illness, and available financial support, such as car park tokens and benefits: “Forewarned is forearmed, isn’t it” (P3). Participants believed they would have also benefited from advice on how to approach their employer and relevant legislation, as well as cheaper alternatives for the meal plan.

### Things that helped

This theme represents participants accounts of factors that eased their situation and helped to manage the difficulties associated with ED caregiving.

#### Tag team

Participants described sharing caring duties with their partner, which was particularly important during periods that their child required constant supervision. This arrangement was also crucial to maintaining financial stability, employment, and emotional well-being: “I’ve been fortunate that I’ve had somebody when I’ve struggled. Who’s been able to take over, or you know, pick me up and then vice versa when it’s impacted emotionally on him, I’ve been able to hold” (P11). The participant from a single-parent household discussed the challenges of coping with a child in hospital, whilst having another child to care for. Other participants reflected on how difficult the situation would have been without their partner’s support: “it’s got to be a joint front … it must be incredibly difficult to be doing it by yourself. Having that pressure” (P2).

#### Not every employer will do that

Most participants cited job flexibility as imperative in coping with demands: “if you had like a nine to five job, which meant you were kind of out of the house, I don’t know how you would manage a young person with an ED” (P5). They considered themselves fortunate if they were eligible for paid leave, able to work from home, afforded flexibility with hours, and able to leave for appointments. Some participants expressed gratitude for being able to take unpaid leave whilst retaining employment. Consideration was given to how societal lack of ED understanding can affect interactions with employers: “if people are not aware of how the condition impacts families [and].… what is needed to get better from it …. HR’s [human resources’] approach to it, that’s not going to get better” (P2).

#### It is helpful knowing that they are there

When discussing the ED service, participants expressed appreciation for the comprehensive and extensive support provided, effective communication and cohesion within the team, and being assigned to a consistent clinician. They were grateful for the flexibility of clinicians, who promptly replied to messages with advice, worked to find convenient times for appointments, and explained treatment rationales. The concept of feeling contained was implied throughout participants’ conversations, which helped them through this challenging time: “you can feel in despair sometimes …. I’d always come out of an appointment feeling better than when I went in … just knowing that they’d capture her, and she wouldn’t die” (P8). Participants expressed concerns about accessing support out-of-hours and relying on general services without ED expertise, such as crisis lines or emergency departments.

#### Advice

Participants advised other parents to find out about available support, entitlements regarding time-off work, and financial aid early on. Some suggested applying for DLA or Personal Independence Payment (PIP), which are both non-means-tested benefits available in the UK. Foodbank vouchers were also cited as helpful: “our daughter being fixated on branded foods… if you go to the food bank and get your fruit and your vegetables and save that money and go and buy the branded food” (P9). Advanced planning of meals and shopping were considered important, and participants advised adjusting family meals according to the treatment plan. Additionally, bulk buying, batch cooking, and freezing meals saved both time and money: “I have all, like, ready made things that she can have … That’s made things cheaper” (P7). Participants endorsed parent support groups and forums found through Facebook, and the websites of Eva Musby (https://anorexiafamily.com) and Beat (https://www.beateatingdisorders.org.uk).

### Member reflections

Member reflections were sought from four participants that expressed an interest in reviewing the findings [47]. All participants expressed that the themes and sub-themes accurately reflected their experiences. One participant commented “words are so inadequate at capturing the devastating impact that is ED” (P12). It was agreed collaboratively with this participant to include the following reflection:

Unless you have lived it, there is no table or research paper or book, that can fully encapsulate the trauma of having a child with ED, which continues to ripple years after the NHS have ceased to be able to offer help. (P12)

## Discussion

This is the first study to qualitatively explore how the financial burden associated with caring for a child with an ED is experienced and managed by families. The research indicates that the financial strain can exacerbate the already substantial caregiver burden and interfere with treatment. It also highlights that affordability is often not addressed by services or clinicians, resulting in families feeling unable to discuss financial barriers during appointments and having a limited understanding of available support. The findings provide practical and easily actionable strategies for both parents and services to reduce the negative impact of financial challenges on families and recovery.

As found in a sample of carers in New Zealand [19], participants reported significant psychosocial and economic impacts associated with caregiving. Financial impacts recounted by participants are supported by previous reports examining costs faced by ED caregivers [49]. A report by PricewaterhouseCoopers [7] predicted that carers in the UK spent on average an additional £2800 annually and lost £5950 due to time off work and education. Although parents’ estimates of lost income were lower in the current sample, this may be explained by the COVID-19 restrictions in operation at the time, resulting in many participants working from home [50]. In line with Obeid and colleagues [5], home working conditions were reported to ease the burden in this study; however, other research has suggested the pandemic exacerbated caregiver burden [51, 52].

Despite many parents working from home, all participants reported at least one parent needing to take time off work due to their child requiring constant supervision. Research suggests that parents take on average, 14 weeks off work to support recovery and 14% reported either having to stop working, or losing their job, due to the illness [53]. Time off work appeared to impact one-parent families more, however, only one participant in this study fell into this category and, therefore, the finding must be considered tentative.

Gatt and colleagues [20] proposed that economic hardship and cost-related nonadherence to treatment could contribute to illness maintenance. In this study, two parents expressed that, despite making other sacrifices, they were not always able to afford all treatment components, such as food and transportation to appointments. This suggests that financial barriers may contribute to missed appointments, which are associated with poorer clinical outcomes [54–56]. Additionally, the inability to afford food items required for a high-calorie, nutritious meal plan, may hinder weight restoration, which is considered critical for the recovery of children and adolescents [57]. Many participants also reported that the financial burden adversely affected their mental health. Financial pressures, therefore, intensified the caregiver burden, which can inadvertently maintain ED symptoms [58, 59]. Based on current findings, it is suggested that interventions should clearly inform parents of potential requirements (e.g., time off work and costs), available financial aid, and support conversations with employers and benefit applications.

While some support is available through the UK benefit system for parents caring for a child with a health condition, many parents were unaware of these options, and the amounts received are often minimal [60]. DLA, and PIP for children over 16, are not means-tested and payments range from £28.70 to £108.55 per week. Universal credit may be available to those required to give up work, with a single parent typically receiving around £393.45 per month, or £617.60 for a couple. Carer’s Allowance offers £81.90 per week for those who care for someone for at least 35 hours a week, earn less than £151 per week, and meet other eligibility criteria. The NHS Healthcare Travel Costs Scheme allows individuals referred for specialist treatment to claim a full or partial refund of reasonable travel costs if they receive a means-tested benefit or qualify for the NHS Low Income Scheme, which has similar criteria. Parents in the UK are legally entitled to take a *reasonable* amount of time off work to manage an emergency involving a dependent. They are also entitled to up to four weeks of parental leave per year to care for their child, with 21 days’ notice. However, the law does not require employers to pay for either type of leave, and employers may postpone parental leave for up to six months if it would cause disruption to the business [61].

When providing advice for other parents, participants emphasised the importance of practical considerations. Parents reported primarily relying on informal networks or social media forums for information. However, research indicates parent-led peer support groups could be an effective platform to exchange support and advice [62, 63]. Additionally, research has found that decreases in parental perceived isolation are associated with improvements in their child’s body mass index and psychological and social functioning [64]. Thus, connecting parents of children with EDs could have multiple benefits, especially if grouped according to specific diagnosis or illness duration [62, 65].

Intervening at the early stages of an ED has been widely associated with improved outcomes [66]. Findings suggest psychoeducation is required at a societal level to facilitate earlier intervention, as poor ED understanding among parents and GPs was associated with detection delays [67, 68]. Increased ED awareness also has the potential to improve parents’ overall experiences, as a lack of understanding within the community and social networks can increase burden and feelings of isolation [69, 70]. Parents in the current study were hopeful that increased understanding around EDs and their impact, at a societal level, could also have positive consequences on experiences with employers.

### Limitations

A main limitation with the current research is sampling bias. In line with research aims, participants were only invited to take part if they self-identified as financially impacted by the ED, meaning there may have been over-representation of parents who felt especially burdened. However, given parents reported shame in relation to being unable to afford treatment costs, potential participants may have been dissuaded. Additionally, volunteer samples are likely to consist of parents with the capacity and resources to engage, which may explain why most parents in the current study were from two-parent households and self-reported as financially stable. Practical barriers to participating [71], such as childcare and unsociable working hours, are likely to be exacerbated in this population and are relevant to the findings.

The homogeneity of the sample is also a limitation of the study. Most participants reported being financially stable or comfortable, and owning their own property, which is not representative of the wider population. Individuals facing economic hardship will experience the financial impacts differently, which was highlighted by a small number of participants in this study. Therefore, when interpreting the findings, it must be considered that a diverse range of voices have not been included. This is also relevant in relation to excluding participants who did not have a good level of spoken English and families experiencing high levels of distress. Due to time and resource constraints of the project, it was not possible to translate the necessary materials or recruit a translator for the interviews. Families experiencing high distress were not invited to participate, in line with ethical guidelines, as discussing financial burdens, a known source of distress [30], could have exacerbated their difficulties. It should also be noted that the data does not consider the experiences of those who have not been able to access treatment [72].

Although the sample size was appropriate for this project [33], only a small number of parents’ voices were heard, and all of these had a child currently in treatment at the same service. Therefore, their experiences, particularly those in relation to services, may only be applicable to this ED team or geographical area.

### Future research

Future research should be conducted in other geographical regions, both within the UK and globally. The financial burdens faced by families affected by EDs will vary significantly depending on the available services and healthcare systems in place. Socioeconomic factors should receive greater attention in the ED literature, particularly in clinical trials. Current protocols for evaluating FBT in young people with EDs lack consideration of the potential influence of socioeconomic factors [73]. Further research exploring experiences of parents from a range of family structures is necessary to establish whether the burden is experienced differently by one-parent families. Although this study highlighted financial implications exist, quantitative research should investigate the extent of the impact and better understand who is most affected. Additionally, efforts should be made to include the voices of underrepresented groups [71], particularly those facing practical barriers to participation and those who have not been able to access assessment and treatment due to factors such as social stereotyping and higher body weight [74]. In line with the recommendations provided, interventions should be developed and evaluated for their effectiveness in reducing the financial burden for families and improving quality of life.

### Clinical recommendations

In line with recommendations made by Lock and Le Grange [75], professionals should consider families’ circumstances, including financial situation and family structure, when planning treatment. Services should also routinely address financial implications, and help available, in the assessment and treatment process. This should include information on benefits, reclaiming parking and public transport costs, and accessing foodbank vouchers. However, this needs to be addressed sensitively as parents often feel shame and stigma around not being able to afford requirements.

Services should also consider parental needs and tailor support to reduce caregiver burden. Parents in this study talked favourably about speaking with other parents but also highlighted burdens associated with attending multiple appointments. Research suggests offering groups [76] and appointments [77] online could reduce the burden of travel and associated costs. Studies indicate virtual appointments later in treatment can be effective and serve as a gradual reduction when working towards discharge [78]. However, services must ensure families have the resources to facilitate this.

## Conclusion

This study highlighted that the financial pressures associated with caring for a child with an ED intensify the substantial caregiver burden and, without adequate financial support, some families struggle to cover treatment costs. Findings largely support previous research on parents and carers experiences, in that they face pervasive psychosocial and economic impacts. However, ED services may be able to help reduce the burden by addressing financial challenges during the assessment and treatment process. Practical strategies recommended for ED service providers include offering foodbank vouchers, providing information on available financial support, and tailoring interventions to the unique needs of each family. Advice for other parents, emphasises the importance of learning about available support and entitlements early on, identifying useful online resources, and adopting strategies, such as bulk buying and batch cooking, to save both time and money.

## Data Availability

The data used and analysed during the current study are available from the corresponding author upon reasonable request.

## Abbreviations

ED: Eating disorder
NHS: National Health Service
FBT: Family–based treatment
EDQ: Exploratory descriptive approach
EBE: Expert–by–experience
RTA: Reflexive thematic analysis
GP: General practitioner
DLA: Disability Living Allowance
HR: Human resources
PIP: Personal Independence Payment
